# Siddha COVID Care Centers in Tamil Nadu, India: Coverage, Workload, and Knowledge Gaps

**DOI:** 10.7759/cureus.78369

**Published:** 2025-02-02

**Authors:** Shanmugasundaram Natarajan, Chandrasekaran Anbarasi, Muthappan Sendhilkumar, Elumalai Rajalakshmi, Sasirekha Ranganathan, Siddique Ali T Rahamathullah, Sasikumar Devasenapathy, Pitchiahkumar Murugan, Manickam P

**Affiliations:** 1 Central Council for Research in Siddha, Ministry of Ayush, Government of India, Thiruvananthapuram, IND; 2 Clinical Research, Siddha Regional Research Institute, Thiruvananthapuram, IND; 3 Integrative and Complementary Medicine, Central Council for Research in Siddha, Chennai, IND; 4 Integrative Medicine, Indian Council of Medical Research, Chennai, IND; 5 Epidemiology and Public Health, Tamil Nadu Centre of Excellence for Advanced Manufacturing (TANCAM), Chennai, IND; 6 Integrative and Complementary Medicine, Government Siddha Medical College, Directorate of Indian Medicine and Homoeopathy, Government of Tamil Nadu, Chennai, IND; 7 Epidemiology and Public Health, Indian Council of Medical Research-National Institute of Epidemiology (ICMR-NIE), Chennai, IND

**Keywords:** coivd-19, coronavirus, indian traditional medicine, siddha, siddha covid care centre

## Abstract

Introduction

The COVID-19 pandemic has significantly impacted India since March 2020. The Siddha system of medicine is one of the oldest medical systems and originated in Tamil Nadu. It is popular among countries where Tamil-speaking populations live. In recent years, it has controlled epidemics like dengue and chikungunya in Tamil Nadu. Siddha COVID Care Centers (SCCCs) were established in Tamil Nadu to support COVID-19 management. This study assesses the coverage, workload, and gaps associated with these SCCCs.

Methods

A cross-sectional study was conducted in June-July 2021, including all SCCCs, with five patients selected from each center. Descriptive statistics were used to analyze facilities, patient demographics, and medical provisions, focusing on hygiene practices, patient care, and Siddha treatment utilization. Patient adherence to preventive measures was also assessed.

Results

Data were collected from 46 SCCCs, involving 155 patients and 46 physicians. Most SCCCs were located in educational institutions like colleges, schools, or public utility halls like marriage halls and indoor auditoriums (n = 43; 93%). Essential resources such as tap water (n = 30; 65.2%), hand sanitizer (n = 34; 73.9%), and soap (n = 33; 71.7%) were available. Most centers had infrared thermometers, pulse oximeters (n = 41; 89.1%), and BP apparatus (n = 44; 95.6%). Trained Siddha physicians were present in most centers (n = 46; 100%). By June 2021, 15,744 patients had been admitted, with 929 referred to other facilities and 287 discharged against medical advice.

Conclusions

The study highlights the comprehensive approach of SCCCs, incorporating Siddha medicine, dietary interventions, and psychological support. While effective, challenges included resource limitations and the need for better integration of traditional medicine in future pandemic responses. These findings provide valuable insights for public health authorities considering Siddha medicine in pandemic strategies.

## Introduction

COVID-19 has had a profound impact on the Indian population and economy since March 2020 [1]. In response to this pandemic, every state in India implemented public health strategies recommended by the World Health Organization (WHO), including testing, isolation, and contact tracing. Individuals were prioritized for testing to isolate infected persons [2]. Upon identifying positive COVID-19 cases, a protocol from triage to hospital care was followed. The government of India notified guidelines for managing COVID-19; accordingly, symptomatic cases over 55 years old with comorbidities were hospitalized in designated COVID-19 hospitals. The asymptomatic cases under 55 years old without comorbidities were admitted to designated COVID care centers (CCC), which are state-established facilities for isolating and monitoring COVID-19 individuals [3,4].

During the COVID-19 pandemic, the public and private healthcare systems were overburdened. The effective management of COVID-19 was hindered by the lack of specific knowledge regarding its transmission, which was initially believed to be primarily through respiratory droplets. Furthermore, triage strategies were necessary to prioritize patients in need of critical care as the healthcare system struggled with the increase in COVID-19 cases. With its documented role in addressing other viral conditions, such as dengue, traditional medicine has provided valuable insights and alternative approaches to alleviate the burden on conventional healthcare resources [5]. In addition, traditional medicine serves as a complementary healthcare system that supports public health initiatives during times of crisis. Therefore, acknowledging and integrating traditional medicine into the larger healthcare framework becomes essential.

Traditional medicines have also been considered potential drugs for COVID-19, citing the ethical framework released by the WHO on the Ebola virus called Monitored Emergency Use of Unregistered Interventions [6]. The Siddha system of medicine, which originated from Tamil Nadu and is popular among Tamil-speaking populations, has contributed to controlling epidemics like dengue and chikungunya in recent times through the Directorate of Indian Medicine and Homeopathy (DIMH) [7,8].

Efforts to control COVID-19 in Tamil Nadu included the distribution of Kabasura kudineer and Nilavembu kudineer and a unique program called “Arokkiam” launched to include Siddha medicines in the management of laboratory-confirmed mild cases. The DIMH established the Siddha CCCs (SCCCs) throughout the state to provide facility-based isolation and monitoring of COVID-19 individuals through the Siddha system of medicine. Guidelines were issued for establishing and operating COVID-19 care centers, typically set up in educational institutions and public utility facilities such as marriage halls and community halls, with each SCCC attached to a designated COVID-19 hospital [9]. During the first wave of COVID-19, over 29,000 COVID cases were managed and discharged from 29 SCCCs, and more than 54 new Siddha COVID-19 care centers were set up till June 2021 during the second wave of COVID-19.

While SCCCs were implemented during this COVID-19 crisis and attraction toward Siddha medicines among the public increased, the Siddha system of medicine is not fully incorporated into the public health services of the Government of Tamil Nadu [10]. The historical role of Siddha medicine during cholera epidemics was sparsely documented by a few authors in textbooks without any scientific publications on the subject [11]. However, this pandemic has created an opportunity to explore and utilize traditional Siddha medicine practices in managing COVID-19. Hence, this study aimed to evaluate the preparedness and functionality of SCCCs in Tamil Nadu during the COVID-19 pandemic and to identify gaps in resources, infrastructure, and practices to improve future pandemic preparedness using Siddha medicine. We describe the operations, coverage, workload, and knowledge gaps of SCCCs.

## Materials and methods

Study design and setting 

We conducted a cross-sectional study between June and July 2021 in all SCCCs providing treatment for COVID-19 cases in Tamil Nadu. We observed and documented the functionality of SCCCs in terms of amenities, equipment, infection control, registers, treatment facilities, staff training in handling COVID-19, and referral mechanisms.

Inclusion criteria

Asymptomatic cases who were not experiencing any symptoms and had oxygen saturation at room air of more than 93% and clinically assigned mild cases with upper respiratory tract symptoms with or without fever, without shortness of breath, and had oxygen saturation at room air of more than 93% were included in the study [12].

Sampling and sample size

No sampling was done; we included all the functioning SCCCs. We conveniently selected five willing patients from each SCCC for interviews. We also included all the staff present during the visit.

Data collection

The investigators administered a semi-structured questionnaire to assess the operations of SCCCs. We used qualified investigators to collect the data from the patients and staff at SCCC. We interviewed all the staff members present at the SCCC to gather information about the training of the health staff on handling SCCCs, monitoring of the patients admitted in the SCCC, services provided to the patients in SCCC, and protocol for referral/discharge from SCCC. We also used a checklist to abstract information from registers and records regarding the functionality of SCCCs and the guidelines issued by the DIMH. We performed a descriptive analysis, presenting the results as means and proportions. The collected data were analyzed using Epi Info™ version 7.1.5.

Ethical approval

The study was approved by the Institutional Ethics Committee of Government Siddha Medical College in Chennai, India and registered in the Clinical Trial Registry of India (CTRI/2021/07/034506).

## Results

We collected data from 54 SCCCs run by the Government of Tamil Nadu. We analyzed data from 46 SCCCs with 155 patients and 46 physicians. However, 75 patients could not be included in the analysis because of incomplete data. Most SCCCs were in educational institutions and public utility halls (n = 43; 93.4%). In terms of essential requirements for CCCs, running water taps, soap, and hand rubs for disinfection were provided. Most of the centers were provided with tap water (n = 30; 65.2%), hand rub (n = 34; 73.9%), and soap (n = 33; 71.7%) in their premises. However, some facilities lacked soap (n = 23; 50%) and towels (n = 14; 30.4%) for patients. Most SCCCs used personal protective equipment (PPE) kits (n = 39; 84.7%) and surgical masks (n = 38; 82.6%). However, reusable gloves usage was minimal (n = 6; 13.0%). We also observed that approximately three-fourths (n = 33; 71.7%) of the patients wore masks and maintained social distancing (n = 35; 76.0%). Most SCCCs had infrared thermometers, pulse oximeters (n = 41; 89.1%), and BP apparatus (n = 44; 95.6%). The majority of SCCCs have trained Siddha physicians (n = 46; 100%), staff nurses (n = 33; 72%), and helpers (n = 36; 78%) to provide care. In 78% of the SCCCs, a duty roster plan was in place to limit the number of healthcare workers exposed to patients (Table 1).

**Table 1:** Availability of facilities, equipment, and COVID-19 practices at SCCCs in Tamil Nadu, June-July 2021 (n = 46) PPE, personal protective equipment; SCCCs, Siddha COVID Care Centers

Characteristics	N	Percentage (%)
Equipment		
Infrared thermometer	41	89
Stethoscope	36	78
BP apparatus	40	87
Pulse oximeter	41	89
Oxygen cylinder	22	48
Respirator	31	67
Personal protectives		
Surgical mask	38	83
PPE kits	39	85
Latex gloves	33	72
Reusable gloves	6	13
Disinfectant mask	30	65
Patient kit		
Plain soap	23	50
Paper towels	14	30
Linen bags	16	35
Toothbrush and paste	22	48
Bucket and mug	23	50
Human resource availability		
Doctors in the centers	38	83
Staff nurses in the centers	30	65
Helpers in the centers	29	63
Sanitary workers in the centers	36	78
Practices		
Patients wearing masks	33	72
Staff wearing full PPE in the centers	26	56
Patients practice social distancing	35	76

In Tamil Nadu, 15,744 patients were admitted to SCCCs until June 2021. Of these, 929 patients were referred to higher centers, 287 were discharged against medical advice, and the remaining were discharged normally. At the time of visit to the facility, there were 865 patients, and SpO2 readings were recorded for over 90% of them (n = 828) (Table 2). Sufficient human resources were deployed in the SCCCs (Table 3). The mean age of admitted patients was 38.4, and male patients constituted 54.2%. Basic care, such as daily examinations, distribution of Government of Tamil Nadu-approved Siddha polyherbal decoction - Kaba Sura Kudineer (KSK), and timely provision of food, was provided in almost all facilities. Additionally, two-thirds of the patients received telecounseling (n = 119; 76.8%) (Table 4). The mean age of the physicians was 35.6 years, and a little over half of them worked for more than one week and received online training about the guidelines for the operation of SCCC issued by the DIMH, Government of Tamil Nadu (n = 25; 54%), but one-fourth (n = 12; 26%) only had taken the COVID-19 test. Only about one-fifth of the physicians reported having a designated area to remove their PPE and take a rest (n = 37; 80%) (Table 5).

**Table 2:** Profile of COVID-19 patients at SCCCs in Tamil Nadu, June-July 2021 AMA, against medical advice; SCCCs, Siddha COVID Care Centers

Characteristics	N
Patients’ profile	
Admitted in the SCCCs	15,744
Referred from the SCCCs	929
Discharged AMAs	287
Patients on the day of the visit	865
Patients Spo2 recorded	828

**Table 3:** Profile of human resources at SCCCs in Tamil Nadu, June-July 2021 (n = 46) SCCCs, Siddha COVID Care Centers

Human resources in the centers	N	Percentage (%)
Doctors	46	100
Nurses	33	72
Helpers	36	78
Sanitary workers	41	89

**Table 4:** Characteristics of the patients admitted at SCCCs in Tamil Nadu, June-July 2021 (n = 155) KSK, Kaba Sura Kudineer; SCCCs, Siddha COVID Care Centers

Characteristics	N	Percentage (%)
Age of the patient	42 (median)	18 (IQR)
Male	84	54
Examined daily	154	99
Received the masks	102	65
Received awareness	147	94
Received the telecounseling	119	76
Received KSK three times/day	154	99
Comorbidities	32	20
Checked by the doctors	153	98
Received the food three times	154	99
Received the food on time	155	100

**Table 5:** Characteristics of the health care workers and availability of facilities for healthcare workers at SCCCs in Tamil Nadu, June-July 2021 (n = 46) PPE, personal protective equipment; SCCCs, Siddha COVID Care Centers

Characteristics		N	Percentage (%)
Age	Mean	35.6	
Gender	Male	11	24
Comorbid conditions		5	11
Duration of posting	One week	25	54
	One month	11	24
	More than one month	6	13
Received the training		25	54
Done the COVID-19 test		12	26
Provided N-95 masks		37	80
Using PPE kit		36	78
Provided table and chairs		43	93
Place to remove the PPE		37	80
Place to take the rest		37	80
Provided the cots and mattress		36	78
Available drinking water facility		44	96
Available dining place		32	69
Hygienic work environment		44	95

## Discussion

At the very start of the COVID-19 pandemic, the Tamil Nadu state in South India responded by setting up SCCCs for mild and moderate COVID-19-confirmed individuals through the state’s traditional medical system called Siddha medicine. Most SCCCs were located in educational institutions and public utility halls, and most of the centers had all essential items and PPE. We also observed that patients wore masks and maintained social distancing. Regarding human resources, most SCCCs have assigned specifically trained Siddha physicians to provide care. Most cases admitted in SCCCs were asymptomatic and were discharged normally without developing any symptoms. The KSK was provided in a 30 ml dose two times a day, in almost all facilities on time. We documented that most SCCCs followed the guidelines for operating isolation facilities and recorded the profile of patients admitted, referred, and discharged in SCCCs. The majority were discharged normally after 14 days of treatment.

Despite India’s preparation, COVID-19 has imposed a heavy burden on the healthcare sector across India. It has inferred a more significant pressure on healthcare management and its response to the pandemic crisis [13]. India, renowned for its traditional medicine, made efforts to utilize its system to manage COVID-19. Indian systems of medicine are underutilized in public health programs, but COVID-19 has made it unavoidable. COVID-19 caused fragility in health systems worldwide and caught unawares. The role of traditional medical systems in public health emergencies was underestimated. Many countries, including India, South Korea, and China, have incorporated traditional medical systems into mainstream healthcare during COVID-19 management [14]. In South Korea, community treatment centers have been successfully established for the early detection, control, and management of COVID-19 [15].

The Government of Tamil Nadu pioneered setting up SCCC using different technical approaches for effective isolation [9,12]. We documented that most SCCCs were established in educational institutions and public utility halls. The DIMH under the Tamil Nadu government gained experience setting up temporary COVID-19 care centers during the first wave of COVID-19. Setting up educational institutions was appropriate as separate classrooms could be suitably modified for the approach. Public utility halls were also used as a temporary alternative if appropriate educational institutions were unavailable. Additionally, housing board apartments with isolated houses were converted into SCCCs.

SCCCs had ample human resources, including Siddha physicians, staff nurses, multi-tasking staff, and housekeeping staff. However, engaging human resources in public health emergencies proved to be complicated. The state directorate made them available wherever needed. However, some SCCCs lacked skilled human resources. This maiden experience in tackling public health emergencies threw many challenges, including training for handling public health emergencies, and the legacy might be helpful in any future instances. Preventive measures, such as the use of facial masks (triple-layered or N95), hand sanitizer, and PPE, have been widely adopted worldwide to manage COVID-19 [16]. Health workers involved in COVID-19 patient care were provided with these items, as outlined in guidelines issued by the DIMH. However, we documented that not all of the health workers used them, and the use of reusable gloves was specifically worse off. We also identified that one-third of the patients were not wearing masks during the survey. While disinfectants like hand sanitizer and soap were provided in nearly three-fourths of the SCCCs, the shortage of supplies is a critical issue that increases the risk of infection [17]. Many health workers were infected with COVID-19, emphasizing the need to ensure adequate supplies of PPE for all health workers. In addition, the provision of running water taps for hand washing in front of SCCCs was limited due to administrative and technical problems in setting up the facilities in educational institutions and public utility halls.

SCCCs were set up as specialized facilities to provide comprehensive care and support to COVID-19 patients using the Siddha system of medicine without any modern medical intervention. The SCCCs adhered to the guidelines and provided various treatments and therapies to help COVID-19 patients get better care. These centers were staffed by trained and experienced Siddha practitioners who provided personalized care and attention to each patient. They used a combination of Siddha medicines, dietary interventions, yoga and meditation, and other Siddha Varmam therapies to strengthen the patient’s immune system and promote overall wellness. One of the key features of SCCCs is the emphasis on early intervention and proactive treatment. Patients were closely monitored and evaluated from admission to ensure their condition was stable and improving. Any signs of deterioration were immediately addressed, and appropriate measures were taken to prevent further complications. In addition to medical care, SCCCs offered psychological and emotional support to patients and their families. This was particularly important in the context of COVID-19, which had a significant impact on a person's mental health and well-being [18].

Although SCCCs are equipped with personal protective gear and necessary resources to manage COVID-19, the study found that the lack of human resources and the facilities in public utility halls and educational centers were slightly cumbersome. In many centers, physicians were spending out of their pocket to provide extra nutritional dietary supplements, herbal soups, etc. While they have done their job efficiently, there is a need to build proper integrative medicine for handling such pandemics.

Limitations

The study had two limitations. First, we intended to include 230 patients overall, with five patients from each SCCC; however, due to incomplete data, 75 patients had to be excluded from the analysis. This reduction in sample size may affect the generalizability of the results. The incomplete data could have been due to various factors, such as missing information in medical records and difficulties in data collection across multiple SCCCs. Second, asymptomatic individuals experienced agitation and distress due to their perception of being unnecessarily confined in isolation centers. This emotional state led to a purposive selection of willing patients at each SCCC rather than a truly random sampling.

## Conclusions

SCCCs have demonstrated a comprehensive and innovative approach to managing the pandemic through traditional Siddha medicine. This study has highlighted the implementation of guidelines and the overall preparedness of SCCCs in combating COVID-19. While the centers have shown promise in providing holistic patient care, some areas for improvement have been identified. These findings underscore the potential of the traditional Siddha medical system to play a significant role in future pandemic responses. Additionally, policymakers should consider incorporating successful elements of the SCCC model into healthcare frameworks to enhance overall pandemic preparedness and response capabilities.

## Appendices

Appendix A

**Table 6:** Observation checklists

Q No	Items for observation	Observation
A	Patient disinfection at the entrance {Record “Yes” or “No” based on the availability of the items listed below}	
1	A hand-washing tap is available at the entrance	Yes / No
2	Running water supply available	Yes / No
3	Soap/hand wash solution available	Yes / No
4	Three-layered masks are kept at the entrance forpatients	Yes / No
B	Nursing station {Record “Yes” or” NO” based on the availability of theitems listed below}	
5	A dedicated nursing station is present in a segregatedplace	Yes / No
6	The nursing station is equipped with a couch forexamining patients	Yes / No
C	Equipment at the nursing station {Record the exact count of each item listed below}	
7	Infrared thermometer	
8	Stethoscope	
9	BP apparatus with a Blood pressure cuff	
10	Pulse oximeter	
11	Sharp disposal containers	
12	Oxygen cylinder	
13	Gloves – Latex disposable	
14	Gloves (re-usable)	
D	Logistics for infection control for staff {Record the exact count of each item listed below}	
15	Surgical Mask	
16	Respirator (N95 / FFp2 / equivalent)	

17	Gown and apron (single-use long-sleeved fluid- resistant)	
18	PPE Kit	
19	Alcohol-based hand rub	
20	Collection containers for used equipment	
E	Premises and patient care {Record “Yes” or ”NO” based on the availability of theitems listed below}	
21	Inpatient room, Windows in opposite walls for cross ventilation	Yes / No
22	Are single occupancy rooms available for patients?	Yes / No
23	If shared occupancy, how many patients may beadmitted in one room?	Yes / No
24	If shared rooms, the beds are separated by at least 1 metre	Yes / No
25	Is the toilet facility separate for each room?	Yes / No
26	Availability of Nilavembu Kudineer or KabasuraKudieer for distribution	Yes / No
27	Availability of IEC material for COVID in the premises	Yes / No
F	Patient Kit {Record the exact count of each item listed below}	
28	Plain soap	
29	Paper towels	
30	Linen bags	
31	Multi-Vitamin tablets	
32	Zinc tablets	
G	Environmental disinfection {Record “Yes” or” NO” based on the availability of theitems listed below}	
33	Disinfection solution for environmental cleaning – 10 Litres	Yes / No
34	Power sprayer (Disinfection sprayer)	Yes / No
35	Knapsack sprays(Disinfection sprayer)	Yes / No
36	Small hand pumps(Disinfection sprayer)	Yes / No
H	Staff availability {Record the exact count of each item listed below}	
37	Availability of duty roster	Yes / No
38	No. of doctors present	
39	No. of staff nurses present	
40	No. of attenders present	

41	No. of helpers present	
42	No. of sanitary workers present	
43	No. of police personnel present	
I	PPE practices {Record “Yes” or ”NO” based on the availability of the items listed below}	
44	All the patients are wearing masks	
45	All the staff are in full PPE	
46	Observed patients are maintaining social distancing	

Appendix B

**Table 7:** Data abstraction form

Q No	Information on	Response
A	Since the centre was operational (Record date):	dd:mm:yy
1	No. of patients admitted	
2	No. of patients referred to hospital	
3	No. of patients discharged AMA	
B	Statistics in the last 14 days	
4	Total patients admitted	
5	Total patients discharged	
6	Total patients referred to hospitals	
7	Total patients discharged AMA	
8	No. of patients with SPO2 documented among referral	
9	No. of patients with blood pressure documented amongreferral	
10	No. of patients with temperature documented among referral	
11	No. of patients with provisional diagnosis documented amongreferral	
C	Statistics on the day of the visit	
12	Total patients present in the centre	
13	No. of patients for whom SPO2 is recorded	
14	The average number of times SPO2 was measured	
15	No. of doctors posted per day as per the roster	
16	No. of staff nurses posted per day as per the roster	
17	No. of attenders posted per day as per the roster	
18	No. of helpers posted per day as per the roster	
19	No. of sanitary workers posted per day as per the roster	
20	No. of police personnel posted per day as per the roster	

Appendix C 

**Table 8:** Questionnaire for interviewing the patients in Siddha COVID Care Centers

Q No	Questionnaire	Response
1	What is your age (in completed years)	
2	What is your gender?	1. Male 2. Female
3	Date of admission to the COVID care centre	dd:mm:yy
4	How many hours did it take you to enter yourallotted room after reaching this centre?	
5	What is the maximum number of patients that you have shared your room with until now?	
6	Were you examined by the doctor daily?	1. Yes 2. No
7	How many surgical face masks were given toyou at the SCCC at the time of admission?	
8	How many surgical face masks you havecurrently?	
9	Do you receive any awareness messages from the health team	Yes/No
10	Were you given counseling for COVID by healthteam / over the telephone?	Yes/No
11	How many multivitamin tablets were given at admission?	
12	How many Zinc tablets were given atadmission?	
13	Are you suffering from any other medical condition?	1. Yes 2. No
14	What is the other medical condition?	
15	Did you get any health-related issues duringyour stay here?	1. Yes 2. No
16	Were you been checked by a doctor at SCCC?	1. Yes 2. No
17	Do you get food all three times a day?	1. Yes 2. No
18	Do you get food on time for most of the days?	1. Yes 2. No

Appendix D

**Table 9:** Questionnaire for interviewing the health care provider in Siddha COVID Care Centers

Q No	Indicator	Response
1	What is your age (in completed years)	
2	What is your gender?	1. Male 2. Female 3.Transgender
3	What is your designation?	
4	What is your parent institution?	
5	Are you having any co-morbid condition?	
6	For how long you are posted in this SCCC?	
7	Were you trained in caring for COVID – 19patients?	1. Yes 2. No
8	Were you tested for COVID-19 since you areposted in this SCCC?	1. Yes2. No
9	Did you use an N95 mask at all the times insideSCCC?	1. Yes 2. No
10	Do you use full PPE while you are at the SCCC?	1. Yes 2. No
11	How many PPEs do you use in one day?	
12	Do you have a dedicated patient treatment room?	1. Yes 2. No
13	Do you have a table and chair for working?	1. Yes 2. No
14	Do you have a place for wearing PPE?	1. Yes 2. No
15	Do you have a separate place for removing PPE?	1. Yes 2. No
16	Do you have a separate place for taking rest inbetween the work?	1. Yes 2. No
17	Do you have a cot with a mattress for taking rest?	1. Yes 2. No
18	Do you have a drinking water facility for yourself?	1. Yes 2. No
19	Do you have an identified place for dining?	1. Yes 2. No
20	Is the working environment hygienic?	1. Yes 2. No

## References

[1] Singhal T (2020). A review of coronavirus disease-2019 (COVID-19). Indian J Pediatr.

[2] (2020). Advisory for coronavirus. http://pib.gov.in/Pressreleaseshare.aspx?PRID=1600895.

[3] Krishnasamy N, Natarajan M, Ramachandran A (2021). Clinical outcomes among asymptomatic or mildly symptomatic COVID-19 patients in an isolation facility in Chennai, India. Am J Trop Med Hyg.

[4] (2023). Ministry of Health and Family Welfare. https://www.mohfw.gov.in/..

[5] Selvavinayagam T (2016). Learning from Chennai floods to mitigate epidemic. Int J Health Syst.

[6] (2023). Emergency use of unproven clinical interventions outside clinical trials: ethical considerations. https://www.who.int/publications-detail-redirect/9789240041745.

[7] Subbarayappa BV (1997). Siddha medicine: an overview. Lancet.

[8] Zysk KG (2009). Some reflections on Siddha medicine in Tamilnadu. Indian J Hist Sci.

[9] (2021). TN govt opens one Ayurveda & 40 Siddha Covid care centres across the state. https://www.pharmabiz.com/NewsDetails.aspx?aid=138931&sid=1.

[10] Sujatha V, Payyappallimana U (2023). Public health and the politics of knowledge: the role of traditional medicine in the management of COVID-19 in two South Indian states. J Health Manag.

[11] Durairasan G (1951). Siddha Principles of Social and Preventive Medicine. Noi Illa Neri.

[12] COVID-19 Tamil Nadu government orders. https://stopcorona.tn.gov.in/covid-19-tn-government-orders/.

[13] Malik MA (2022). Fragility and challenges of health systems in pandemic: lessons from India's second wave of coronavirus disease 2019 (COVID-19). Glob Health J.

[14] Tong T, Wu YQ, Ni WJ, Shen AZ, Liu S (2020). The potential insights of traditional Chinese medicine on treatment of COVID-19. Chin Med.

[15] Choi WS, Kim HS, Kim B, Nam S, Sohn JW (2020). Community treatment centers for isolation of asymptomatic and mildly symptomatic patients with coronavirus disease, South Korea. Emerg Infect Dis.

[16] Ağalar C, Öztürk Engin D (2020). Protective measures for COVID-19 for healthcare providers and laboratory personnel. Turk J Med Sci.

[17] Jayatilleke SK, Mendis DM, Perera TP, Manoj SS, Iresha GK, Buddhadasa P, Hansani HC Use of personal protective equipment by healthcare workers exposed to COVID-19 infection at a tertiary care hospital in a lower middle-income country. Sri Lankan J Infect Dis.

[18] Kanakavalli DK (2020). Proactive steps by the Ministry of AYUSH in mitigating COVID-19: an update on contribution of Siddha. J R Siddha Med.

